# Analysis of adenylate cyclase activity in Japanese children with orthostatic dysregulation

**DOI:** 10.1371/journal.pone.0347431

**Published:** 2026-04-30

**Authors:** Nobuyoshi Sugiyama, Tomoyoshi Komiyama, Kengo Ayabe, Shin-ichi Matsuda, Mayumi Enseki, Mariko Ikegami, Yoshihiro Miyashita, Ayumi Sasaki, Yuka Kitamura, Atsushi Uchiyama

**Affiliations:** 1 Department of Pediatrics, Tokai University School of Medicine, Isehara, Kanagawa, Japan; 2 Department of Pediatrics, JA Kanagawaken Kouseiren ISEHARA Kyodo Hospital, Isehara, Kanagawa, Japan; 3 Department of Clinical Pharmacology, Tokai University School of Medicine, Isehara, Kanagawa, Japan; 4 Department of Cardiology, Tokai University School of Medicine, Isehara, Kanagawa, Japan; 5 Cardiovascular Center, Miyazaki Medical Association Hospital, Miyazaki, Miyazaki, Japan; 6 Department of Life Science Support, Research Innovation Center, University Hospitals Sector, Tokai University, Isehara, Kanagawa, Japan; University of Pennsylvania Perelman School of Medicine, UNITED STATES OF AMERICA

## Abstract

The aim of the study was to clarify the cause of orthostatic dysregulation in Japanese children by analyzing fluctuations in adenylate cyclase activity. Four types of orthostatic dysregulation in Japan include postural orthostatic tachycardia syndrome, delayed orthostatic hypotension, immediate orthostatic hypotension, and vasovagal syncope. However, the exact cause of these disorders remains unknown. To identify the cause of these conditions, we examined the resting blood adenylate cyclase activity and basic clinical data (blood pressure, pulse rate) of 30 patients diagnosed with orthostatic dysregulation (21 postural orthostatic tachycardia syndrome, eight delayed orthostatic hypotension, one immediate orthostatic hypotension, zero vasovagal syncope) and 20 previously reported healthy adults. The results of this study showed that adenylate cyclase activity (isoproterenol and adrenaline) in patients with postural orthostatic tachycardia syndrome was significantly higher than that in patients with delayed orthostatic hypotension and healthy adults. Moreover, patients with postural orthostatic tachycardia syndrome had significantly higher values than healthy adult controls at all five concentration points. Adenylate cyclase activity in patients with delayed orthostatic hypotension showed a trend toward higher values at 10 μM of adrenaline. Furthermore, owing to the higher adenylate cyclase activity in patients with postural orthostatic tachycardia syndrome, their systolic blood pressure was higher than that in patients with delayed orthostatic hypotension. These results suggest that increased adenylate cyclase activity may be related to the onset of orthostatic dysregulation (postural orthostatic tachycardia syndrome and delayed orthostatic hypotension). In conclusion, adenylate cyclase activity levels may be related to the onset of orthostatic dysregulation, and this can be used as a new strategy for diagnosing orthostatic dysregulation.

## Introduction

In the pathology of orthostatic dysregulation (OD), the regulatory mechanism mediated by the autonomic nervous system does not function properly in response to the circulatory dynamic changes that accompany standing. This condition causes physical symptoms, particularly cardiovascular symptoms, such as dizziness, headache, and fainting [1–6]. However, its fundamental cause remains unclear. In Europe, there is no disease called orthostatic intolerance (OI); however, in the United States, OI and orthostatic hypotension (OH) are similar conditions [7]. Tanaka et al. developed the Japanese OD guidelines, which clearly outline the interview process, differential diagnosis, exclusion diagnosis, and OD subtype classification using the new OD test. Currently, in Japan, diagnosis and treatment are mainly performed according to these OD guidelines [8]. In addition, according to them, considering biological functions and psychosocial factors is necessary for OD treatment [9–12]. Several studies have investigated possible causes of OD [13–20]. Fujii et al. (2011) evaluated the change in inferior vena cava (IVC) diameter before and after head-up tilt and reported that IVC diameter increased or decreased [21]. Jacob et al. (2006) reported abnormal function of β2-adrenergic receptors in patients with postural orthostatic tachycardia syndrome (POTS) [22]. Tanaka et al. (1999) measured the serum noradrenaline (NA) response in instantaneous orthostatic hypertension (INOH) and reported central sympathetic nerve suppression [3]. Moreover, OD persists and sometimes recurs during adulthood. Kazuma et al. (2000) reported that the compensatory regulatory mechanism for changes in circulatory dynamics associated with changing body position, such as standing, failed for an unknown reason, reducing orthostatic tolerance and leading to circulatory dysregulation [4]. This can lead to school refusal and depression in adults [9]. The unknown pathology of these diseases, which has a significant impact on quality of life, can also lead to major social problems. Neurally mediated syncope (NMS) is one of the primary causes of fainting in adults. Recently, a relationship between adenylate cyclase (AC) activity and adult NMS has been reported [23,24]. AC converts adenosine triphosphate to the intracellular messenger cyclic adenosine monophosphate (cAMP), and an increase in cAMP levels relaxes the elasticity of vascular smooth cells [23–32]. Differences in AC activity have been reported in adults with NMS compared with healthy individuals, and this is suggested to be one of the causes of NMS. Childhood OD often persists or recurs into adulthood and presents with symptoms similar to those of NMS, such as dizziness, lightheadedness, and fainting.

The aim of the study was to elucidate the etiology of OD. Given the symptomatic similarities between OD and adult NMS, we hypothesized that these conditions share common symptoms, despite differences in onset age. Here, we report our findings on the relationship between OD and resting AC activity.

## Materials and methods

### Ethics statement

This study was reviewed and approved by the hospital’s Institutional Ethics Committees (approval number: 112). Prior to participation, all patients provided written consent for the clinical research, following the approval of the experimental procedure by the relevant ethics committee at the JA Kanagawaken Kouseiren Isehara Kyodo Hospital. Also, the last date of access to the data was April 8th 2025 and authors did not have access to individual participants information.

### Patients with orthostatic dysregulation

Between April 2018 and December 2024, patients visited the Pediatrics Department of JA Kanagawaken Kouseiren Isehara Kyodo Hospital with symptoms of fainting, dizziness, and lightheadedness. Based on the interview and examination findings, OD was suspected. Blood test, 12-lead electrocardiogram, head magnetic resonance imaging, and electroencephalography findings showed no abnormalities. Thus, 30 patients (13 males and 17 females; age at time of examination, 7 years to 15 years; mean age, 13 years) who consented to participate in this clinical trial were surveyed (S1 Table). Based on the criteria of the Japanese Society of Pediatric Psychosomatic Medicine Guidelines (2015), a new orthostatic test (mercury-free automatic blood pressure monitor KM-385OD, Kent Medico Co., Ltd. Saitama, Japan) was performed, and the patients were diagnosed with OD who only had symptoms of being unable to wake up in the morning and whose symptoms improved within approximately 3 months were excluded from this study. Additionally, patients suspected of anemia (Hb 10.0 g/dl or less), dehydration (UN 21 mg/dl or more), electrolyte abnormalities, or liver dysfunction were excluded from our investigation.

Although parental consent could be obtained, it was difficult to obtain assent from the children themselves. Therefore, recruiting healthy pediatric controls was not feasible because of ethical reasons.

### Blood pressure and pulse measurement

The average blood pressure (BP) and pulse rate were recorded thrice in the supine position at rest before blood sampling using an electronic blood pressure device (TERUMO Electronic Blood Pressure Monitor Elemano, Tokyo, Japan).

All examinations were conducted between 9:00 and 11:00 a.m. Blood pressure was measured using a cuff placed on the right upper arm, and cuff size was selected according to each participant’s age and body size. For systolic and diastolic blood pressure assessment, participants were placed in a supine resting position for 10 minutes, after which blood pressure was measured three times. The final systolic blood pressure value was determined using the median of the three measurements. Healthy adult volunteers were also tested under the same conditions [24,29].

Blood pressure was measured on the day of the AC activity assessment. Repeated measurements at separate visits were not feasible for all patients, as some participants refused additional measurements due to concerns about worsening symptoms. Therefore, blood pressure data from the day of AC measurement were analyzed.

### Blood sampling method

The AC activity test was performed after the patients made the recommended lifestyle changes, including increased fluid intake (minimum, 1.5 L/day), and at least 3 months after the diagnosis of OD. In addition, no oral medication was taken on the morning of the AC test, and only water was allowed to be consumed on the test day. After resting in the supine position for at least 20 min, blood was collected by 9:45 AM.

### Measurement of AC activity

A blood sample was obtained by placing an indwelling needle in a vein in the forearm, and after removing approximately 1–2 mL of blood to avoid contamination from prior medications, 8 mL of blood was collected. To prepare lymphocytes, blood was collected in a Vacutainer mononuclear cell isolation tube (BD Vacutainer® CPT™) and centrifuged to isolate the upper lymphocyte layer. Subsequently, the lymphocytes were washed with culture medium (Roswell Park Memorial Institute 1640), platelets removed, and lymphocytes counted. To measure AC activity, the test reagent forskolin (FK) was added to the lymphocytes (10,000 cells) and incubated at 22–25 °C room temperature for 30 min. The amount of cAMP was measured according to the Promega cAMP-Glo Assay protocol [23,24] Adrenaline (AD) was added at concentrations of 100 μM, 10 μM, and 1 μM. Isoproterenol (IP) was added at concentrations of 50 μM, 5 μM, and 500 nM.

### Adjustment of reagent concentrations for assays (adrenaline, isoproterenol, forskolin)

Having analyzed the concentration determination in our previous paper, we referred to it [23,24,29]. To measure AC activity, we examined the concentrations of the additives AD, IP, and FK to observe the reaction in lymphocytes [23,24]. AD is a nonselective agonist of all adrenergic receptors, including the major subtypes α1, α2, β1, β2, and β3 [26,30–33]. IP activates AC for the production of the adrenergic subtype β2 receptor, which promotes the binding of AC and G protein to produce cAMP [31,34,35]. FK activates AC to produce cAMP [23,24,29]. Therefore, prior to measuring the AC activity, we verified the concentrations of AD and IP when added to the lymphocytes.

### Measurement of adrenaline, noradrenaline, and dopamine levels

A portion of the blood was centrifuged to extract plasma, which was then frozen and measured at an external research institute (SRL, Inc., Tokyo, Japan). Catecholamine levels were measured because they mainly affect the brain, adrenal medulla, and sympathetic nerves and act directly on AC.

### Statistical analyses

All data are reported as mean ± standard deviation (SD). Comparisons were performed using t-tests, as appropriate. The analysis was performed using Microsoft Excel 365 MSO (version 2403 Build 16.0.17425.20176). The statistical analysis was performed using Excel, with a t-test applied to determine significant differences, and results expressed as mean ± SD [23,24,29]. The mean variance is expressed as mean ± SD. For the analysis of AC activity, 100 μM FK was considered 100%, and the percentage of 100 μM AD was calculated accordingly.

### Healthy adult volunteers

This study included 20 healthy adult controls [29] recruited from Tokai University School of Medicine between June 2016 and March 2023. The mean age, systolic BP (SBP), diastolic BP (DBP), and pulse rate were 36.1 ± 8.8 years, 108.6 ± 9.4, 66.9 ± 8.8, and 66.5 ± 8.4, respectively. Our previous study demonstrated that AC activity remained unchanged over time [23]. Therefore, we assumed that AC activity remains almost unchanged from childhood and used the results from healthy adult controls for comparison.

## Results

### Clinical and epidemiological analysis

Based on the diagnosis of the new orthostatic test [8], patients with OD were classified as follows: 21 patients with POTS, eight patients with persistent delayed OH (DOH), and one patient with postural hypotension (INOH) (Table 1).

**Table 1:** Background characteristics of patients with OD.

	Age	Systolic BP	Diastolic BP	Heart rate
POTS (n = 21)	12.8 ± 2.0	102.0 ± 8.2	59.9 ± 5.4	69.1 ± 12.9
DOH (n = 8)	11.5 ± 2.1	108.5 ± 8.7	61.6 ± 4.5	76.9 ± 17.1
*p*-value	–	0.04^*^	0.20^*^	0.13^*^

* *p*-value does not include INOH. Each value is expressed by mean ± standard deviation (SD).

OD, orthostatic dysregulation; POTS, postural orthostatic tachycardia syndrome; DOH, Delayed orthostatic hypotension; INOH, instantaneous orthostatic hypertension; BP, blood pressure.

We compared the resting BP between patients with POTS and DOH. The resting SBP, DBP, and heart rate (HR) were measured. We observed no significant differences in DBP or HR between the POTS and DOH groups. Patients with DOH exhibited a significantly higher average SBP (108.5 ± 8.7 mm Hg) compared with those with POTS (102.0 ± 8.2 mm Hg) at rest (*p* = 0.04). In addition, blood tests showed no significant differences in hemoglobin (Hb), hematocrit (Ht), creatinine (Cr), urea nitrogen (UN), and uric acid levels between patients with POTS and DOH (S1 Table).

### Comparison of AC activity in eight patients with DOH and 21 patients with POTS at rest

**Fig 1:**
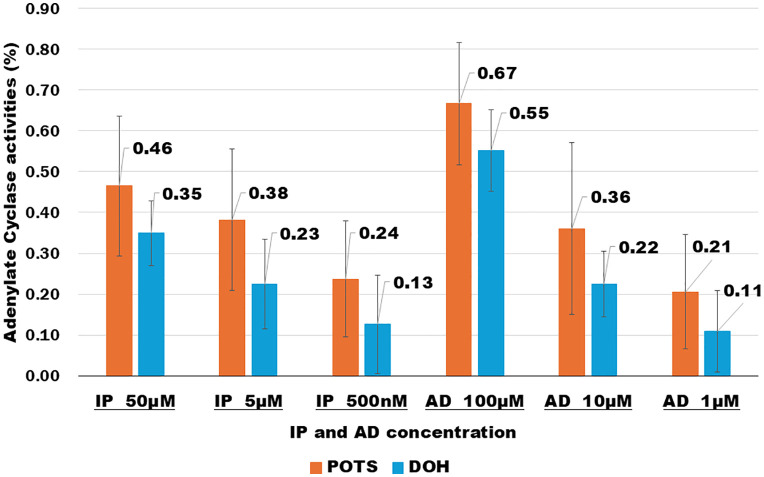
Adenylate cyclase activities in 21 patients with postural orthostatic hypotension and eight patients with delayed orthostatic hypotension.

Next, we investigated the AC activity (IP: 50 µM, 5 µM, 500 nM; AD: 100 µM, 10 µM, 1 µM) at rest between eight patients with DOH and 21 patients with POTS. Significant differences were observed in all comparisons with POTS and DOH at the six concentrations (IP 50 µM: *p* = 0.01, IP 5 µM: *p* = 0.005, IP 500 nM: *p* = 0.03, AD 100 µM: *p* = 0.01, AD 10 µM: *p* = 0.01, AD 1 µM: *p* = 0.02) (Table 2, Fig 1 and S2 Table). Patients with POTS had significantly higher AC activity than those with DOH.

**Table 2:** AC activities (IP and AD) at rest between 21 patients with POTS and eight patients with DOH.

	POTS (%)	SD	DOH (%)	SD	POTS vs. DOH t-test (*p*-value)
IP 50 µM	0.46	0.17	0.35	0.08	0.010
IP 5 µM	0.38	0.17	0.23	0.11	0.005
IP 500 nM	0.24	0.14	0.13	0.12	0.027
AD 100 µM	0.67	0.15	0.55	0.10	0.014
AD 10 µM	0.36	0.21	0.22	0.08	0.009
AD 1 µM	0.21	0.14	0.11	0.10	0.023

SD, standard deviation; AC, adenylate cyclase; IP, isoproterenol; AD, adrenaline; POTS, postural orthostatic tachycardia syndrome; DOH, delayed orthostatic hypotension.

### AC activity in 30 patients with orthostatic dysregulation versus healthy adult controls at rest

**Fig 2:**
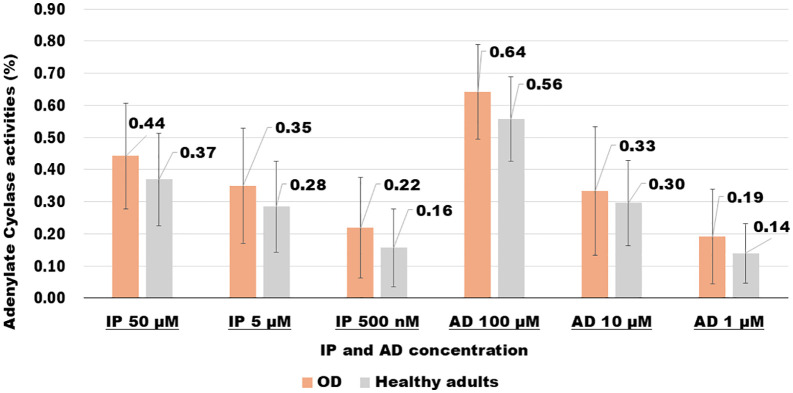
Changes in adenylate cyclase activity in 30 patients with orthostatic dysregulation at six concentrations and 20 healthy adult controls at rest.

We compared the six concentrations (three IP and three AD) of AC activity in all patients with OD with those of healthy adult controls. All concentrations were higher in patients with OD than in healthy adult controls. AC activity was particularly significant: AD at 100 µM in patients with OD was 64% compared with 56% in healthy adult controls. The results of the t-test showed a significant difference in favor of patients with OD (*p* = 0.02). The five other concentrations showed no significant differences (Table 3, Fig 2 and S2 Table). We observed that a higher concentration (AD 100 µM) of the main component led to a stronger reaction in OD.

**Table 3:** Comparison of AC activity in 30 patients with OD at six concentrations and 20 healthy adult controls at rest.

		AC			t-test (*p*-value)
	OD	SD	HACs	SD	
IP 50 µM	0.44	0.16	0.37	0.14	0.05
IP 5 µM	0.35	0.18	0.28	0.14	0.08
IP 500 nM	0.22	0.16	0.16	0.12	0.06
AD 100 µM	0.64	0.15	0.56	0.13	0.02
AD 10 µM	0.33	0.20	0.30	0.13	0.22
AD 1 µM	0.19	0.15	0.14	0.09	0.06

IP, isoproterenol; AD, adrenaline; OD, orthostatic dysregulation; HACs, healthy adult controls; AC, adenylate cyclase; SD, standard deviation.

### AC activity in 21 patients with POTS, eight patients with DOH, and 20 healthy adult controls at rest

**Fig 3:**
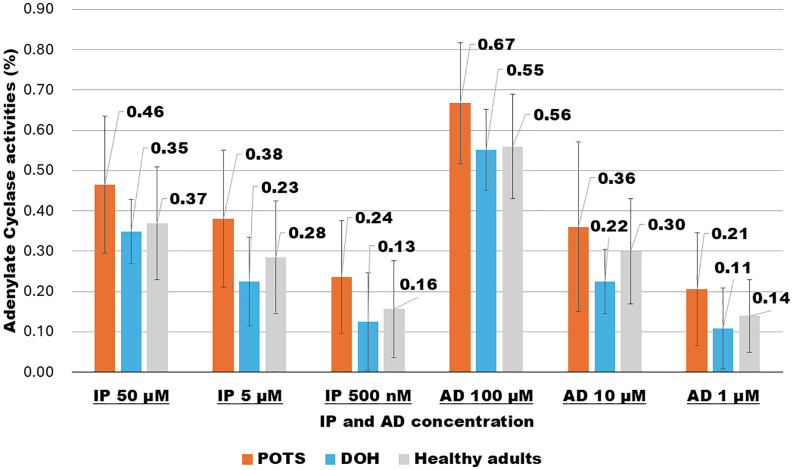
Changes in adenylate cyclase activity at rest in 21 patients with postural orthostatic tachycardia syndrome, eight patients with delayed orthostatic hypotension, and 20 healthy adult controls.

We analyzed patients with OD by dividing them into two groups: POTS (n = 21) and DOH (n = 8) (Table 4, Fig 3 and S2 Table) at rest. One group (POTS) had significantly higher AC activity levels than the HAC group, whereas the other group had lower AC activity levels.

**Table 4:** Comparison of AC activity at rest in 21 patients with POTS, eight patients with DOH, and 20 healthy adult controls.

	POTS	DOH	HACs	POTS vs. HACs t-test (*p*-value)	DOH vs. HACs t-test (*p*-value)
IP 50 µM	0.46 ± 0.17	0.35 ± 0.08	0.37 ± 0.14	0.03	0.32
IP 5 µM	0.38 ± 0.17	0.23 ± 0.11	0.28 ± 0.14	0.03	0.13
IP 500 nM	0.24 ± 0.14	0.13 ± 0.12	0.16 ± 0.12	0.03	0.28
AD 100 µM	0.67 ± 0.15	0.55 ± 0.10	0.56 ± 0.13	0.008	0.45
AD 10 µM	0.36 ± 0.21	0.22 ± 0.08	0.30 ± 0.13	0.13	0.05
AD 1 µM	0.21 ± 0.14	0.11 ± 0.10	0.14 ± 0.09	0.04	0.23

AC, adenylate cyclase; POTS, postural orthostatic tachycardia syndrome; DOH, delayed orthostatic hypotension; HACs, healthy adult controls.

Next, we performed t-tests (*p* < 0.05) for patients with POTS (n = 21). Patients with POTS exhibited significantly higher AC activities with AD (100 μM, 10 μM, and 1 μM), especially at 100 μM (*p* = 0.008) (10 μM, *p* = 0.13; 1 μM, *p* = 0.04). IP at three concentrations (500 nM, 5 μM, 50 μM) showed significantly higher AC activity in patients with POTS than in those with DOH (50 μM, *p* = 0.03; 5 μM, *p* = 0.03; 500 nM, *p* = 0.03). The POTS values were extremely high at all three points, and significant differences were confirmed in all but one test.

### Analysis of adrenaline, noradrenaline, and dopamine levels in the blood

Catecholamines are a general term for biogenic amines that are mainly found in the brain, adrenal medulla, and sympathetic nerves, and three types exist in the body: dopamine, NA, and AD. Therefore, we compared these three types. No significant differences were observed between the POTS and DOH groups (Table 5 and S3 Table).

**Table 5:** Comparison of adrenaline, noradrenaline, and dopamine levels.

	OH		POTS		t-test (*p*-value)
	Average	SD	Average	SD	
Adrenaline	29.13	11.4	36.19	33.6	0.20
Noradrenaline	238.63	70.2	227.81	87.3	0.37
Dopamine^*^	8.13	2.3	8.00	4.6	0.46

* Dopamine levels are ≥ 5. SD, standard deviation; OH, orthostatic hypotension; POTS; postural orthostatic tachycardia syndrome.

## Discussion

This study aimed to investigate the causes of OD in children. To conduct this study, we recruited 30 pediatric patients with OD. Based on the new orthostatic test [8], patients with OD were classified into POTS, DOH, and INOH.

We previously investigated the changes in AC activity in patients with NMS and observed that these changes may contribute to the development of NMS. In this study, we hypothesized that, despite differing onset times, NMS and childhood OD share similar symptoms. Therefore, we examined the relationship between OD and AC activity by measuring resting AC activity in children with OD. We conducted clinical epidemiological studies as previously described. Blood tests revealed no significant differences in Hb, Ht, Cr, UN, or uric acid levels between patients with POTS and DOH. Although dehydration has been associated with both POTS and DOH, no blood test abnormalities were observed in patients with OD. No pathological conditions that caused blood test abnormalities were identified. While such as Zhong Xi Yi et al. (1990) have reported the association between Hb and red blood cell values and OD symptoms, our study excluded patients with preexisting blood abnormalities (anemia, dehydration, thyroid dysfunction) [36]. Consequently, we observed no correlation between the OD and blood test results in our patient group.

However, when comparing resting SBP, DBP, and HR between patients with POTS and DOH, SBP was significantly lower in patients with POTS than in those with DOH. Because of the small sample size, the present findings should be interpreted with caution and may change as additional data becomes available. Compared with the American Pediatric Hypertension guidelines [37], SBP was low in all 30 patients, both for their respective age groups and regardless of age, but particularly in those with POTS.

The current study examining the relationship between resting AC activities and BP revealed that resting AC activity was significantly higher in patients with POTS. AC exerts vasodilatory effects. Although we measured resting AC activity in this study, it can be hypothesized that patients with persistently high AC activity have a tendency to develop hypotension due to their condition. Guidelines recommend the use of β-blockers and midodrine hydrochloride (Sawai Pharmaceutical Co., Ltd. Osaka, Japan) for OD treatment. Although their effectiveness is limited, the AC results of this study are consistent with OD pathophysiology [8].

Resting AC activities were 63% in all patients with OD and 54% in healthy adult controls. The results of the t-test showed a significant difference in patients with OD, with a *p*-value of 0.02, and differences in high, equal, and low values were observed from childhood. AC activity in patients with OD was different from that in healthy adult controls. There are differences in AC activity during childhood. Furthermore, Stewart et al. reported that in Europe, the United States, and Japan, 20–30% of individuals with OI/OD continued to experience symptoms into adulthood. They noted that although OI/OD involving syncope often improved in patients in their 20s, it frequently relapsed in middle age [7]. This is a possible cause of the persistence and recurrence of OD symptoms in adulthood. Thus, fluctuations in AC activity are related to OD.

Wittwer et al. (2011), Eduardo et al. (2012), and Jacob et al. (2006) demonstrated that mutations in the beta-1 and beta-2 adrenergic receptor genes in patients with OI or POTS might affect cardiovascular reactivity to orthostatic stress and other stimuli [22,38,39]. Furthermore, the development of primary idiopathic POTS is thought to be related to multiple mechanisms, including viral illness (or any condition that activates the innate immune response); peripheral nerve denervation with hypersensitivity; α-receptor hypersensitivity; autoantibodies to acetylcholine and β-receptors; central hyperadrenergic state; norepinephrine transporter deficiency; reduced baroreceptor gain; idiopathic hypovolemia with changes in aldosterone, renin, neuronal nitric oxide synthase, and angiotensin II activities; autoimmune reactions; mast cell activation; and decreased heart size and mass [38–51]. In OI, particularly POTS, high levels of resting AC activity during childhood may indicate the presence of congenital genetic factors.

Furthermore, we observed differences in AC activity levels based on IP and AD concentrations. This suggests that variations in AC activity are likely influenced by a strong activation response of the B2 receptor and a weak suppression response of ADRa2B. In other words, it is inferred that these differences are due to variations in subunit binding energy obtained from the protein structure analysis of the α2B-AR receptor gene [23,33–35,52–57]. This aligns with the responses observed in our previous genetic analysis of patients with NMS.

In this study, the resting AC activity was lower in patients with initial DOH than in patients with POTS or healthy adults. A tendency toward higher SBP was observed. We were unable to compare the AC activity levels between patients with DOH and POTS. However, if significant differences are observed between these two subtypes, it is expected to further elucidate the pathogenesis of OD. Furthermore, we believe that by referring to the SBP and AC activity values obtained in this study, it will be possible to establish a new method for OD diagnosis. In particular, it was suggested that a concentration of 100 μM AD was effective. The fluctuations in AC activity observed in this study suggest the possibility of transitioning from POTS to vasovagal-type NMS and from DOH to mixed-type NMS (MT-NMS). Interestingly, this study also revealed that AC activity levels in children are already higher or lower than those in adults. This underscores the importance of conducting follow-up studies on patients involved in this study to elucidate their relationship with future NMS.

### Limitations

The AC activity levels in healthy adult controls were measured as described by Komiyama et al. Healthy children were not used as normal comparison participants because there is a possibility that they might develop OD in the future; therefore, healthy adults were used instead. Furthermore, for ethical reasons, we do not include healthy children as participants in our clinical study.

## Conclusions

Patients with DOH have a higher SBP, whereas those with POTS have a lower SBP. Furthermore, our findings indicate a potential association between AC activity levels and BP fluctuations. This suggests that differences in AC activity at rest can serve as supplementary markers for the detailed diagnosis of OD subtypes. This study suggests that variations in AC activity contribute to the onset of OD, potentially leading to the development of new diagnostic methods for this condition.

## Supporting information

S1 Table Characteristics of the 30 OD patients. (PDF)

S2 Table Raw data and t-test for AC activities between DOH, POTS and HAC. (PDF)

S3 Table Raw data of adrenaline, noradrenaline, and dopamine levels in the blood. (PDF)
