# Supplementary material for: Analysis of adenylate cyclase activity in Japanese children with orthostatic dysregulation
Source: PLoS One. 2026 Apr 30;21(4):e0347431. doi: 10.1371/journal.pone.0347431 (PMC13132173; doi:10.1371/journal.pone.0347431)
Supplement: S1 Table — (PDF) [file pone.0347431.s001.pdf]

S1 Table. Characteristics of the 30 OD patients.

	Number	Age	Sex	Systolic BPmmHg	Diastolic BPmmHg	Pulse	Hb (g/dl)	Ht (%)	Cr (mg/dl)	UN (mg/dl	UA (mg/dl
Delayed OH	1		Female	116	68	86	15	43.9	0.39	14	4.2
	2		Male	98	63	60	12.9	38.1	0.44	14	3.4
	3		Male	100	65	72	14.8	42.3	0.59	9	7.1
	4		Male	114	63	81	13.8	40.2	0.61	11	7.8
	5		Female	118	59	113	13.3	38.4	0.35	10	3
	6		Female	114	53	70	13.9	41.6	0.5	11	4.4
	7		Male	111	60	61	12.6	38.1	0.48	11	3.9
	8		Female	97	62	72	12.9	37	0.44	11	3.5
	**Average**	11.50	—	108.50	61.63	76.88	13.65	39.95	0.48	11.38	4.66
	**Standard Deviation**	2.07	—	8.68	4.47	17.06	0.89	2.45	0.09	1.77	1.79
POTS	1		Female	97	55	64	13.8	39.1	0.43	12	3.8
	2		Female	92	50	73	13.5	40.2	0.38	8	4.3
	3		Female	102	65	101	11.2	34.3	0.29	9	3.8
	4		Female	104	66	81	14.1	42.8	0.36	9	5
	5		Male	98	54	59	14	46.6	0.57	11	5.7
	6		Female	103	63	85	13.5	40.2	0.38	8	3.3
	7		Male	91	59	88	13.3	44.1	0.47	8	4
	8		Male	114	67	66	13.3	39.4	0.44	11	4.7
	9		Male	99	53	68	14.1	43.9	0.51	9	5.4
	10		Male	97	52	59	12.6	37.1	0.47	8	4.2
	11		Female	97	58	67	13.1	38.7	0.55	9	4.6
	12		Female	100	60	60	12.7	36.8	0.45	10	4.9
	13		Female	96	54	67	13.6	37.5	0.75	12	5.8
	14		Male	107	65	52	14.3	41.2	0.49	12	3.7
	15		Female	102	66	73	12.2	38.5	0.59	17	4.1
	16		Female	89	60	65	12	36.7	0.56	9	5.3
	17		Male	114	67	54	14.2	44.8	0.85	12	6.6
	18		Male	119	64	87	14.6	42.7	0.46	14	2.8
	19		Female	106	61	66	12.7	37.7	0.47	10	4.7
	20		Male	99	57	51	15	42.9	0.49	14	3.6
	21		Female	115	62	65	14.3	43.4	0.67	11	4.9
	**Average**	12.81	—	101.95	59.90	69.10	13.43	40.41	0.51	10.62	4.53
	**Standard Deviation**	2.02	—	8.16	5.36	12.93	0.94	3.24	0.13	2.38	0.92
INOH	1		Female	102	68	73	13	39.7	0.62	14	4.2
	**Average**	12.50	—	103.70	60.63	71.30	13.48	40.26	0.50	10.93	4.56
	**Standard Deviation**	2.06	—	8.53	5.21	14.06	0.91	2.96	0.12	2.26	1.17
DOH ver. POTS	*t*-test	—	—	0.04	0.20	0.13	0.29	0.34	0.24	0.18	0.43
