# Supplementary material for: Analysis of adenylate cyclase activity in Japanese children with orthostatic dysregulation
Source: PLoS One. 2026 Apr 30;21(4):e0347431. doi: 10.1371/journal.pone.0347431 (PMC13132173; doi:10.1371/journal.pone.0347431)
Supplement: S2 Table — (PDF) [file pone.0347431.s002.pdf]

S2 Table. Raw data and t-test for AC activities in DOH, POTS and HAC.

delayed OH n=8	1	2	3	4	5	6	7	8			DOH vs. POTS
	delayed OH	delayed OH	delayed OH	delayed OH	delayed OH	delayed OH	delayed OH	delayed OH	**Average**	**SD**	**t-test**
Isoproterenol 50uM	0.41299602	0.42005622	0.353553238	0.186361963	0.424584915	0.343157099	0.361852171	0.290912426	**0.349184256**	**0.080091583**	**0.010423611**
Isoproterenol 5uM	0.288091	0.252314408	0.14608388	0.055088362	0.381578542	0.261702054	0.303781903	0.112355051	**0.2251244**	**0.109924324**	**0.004558249**
Isoproterenol 500nM	0.214772613	0.186932045	-0.012442334	0.00312728	0.320131198	0.13357856	0.167842547	-0.004864903	**0.126134626**	**0.120994633**	**0.027202447**
Adrenaline 100uM	0.658629004	0.69287865	0.599298281	0.372855665	0.526321544	0.513203927	0.487785489	0.558504635	**0.551184649**	**0.101327878**	**0.013650417**
Adrenaline 10uM	0.23523051	0.286353672	0.11299194	0.138934322	0.342856084	0.225690454	0.281318407	0.176073205	**0.224931074**	**0.078629449**	**0.009392023**
Adrenaline 1uM	0.07387963	0.169766992	-0.005534061	0.027650991	0.282343798	0.139018374	0.157592144	0.025902041	**0.108827489**	**0.096271331**	**0.023144689**

SD: standard deviation

POTS n=21	1	2	3	4	5	6	7	8	9	10	11	12	13	14	15	16	17	18	19	20	21		
	POTS	POTS	POTS	POTS	POTS	POTS	POTS	POTS	POTS	POTS	POTS	POTS	POTS	POTS	POTS	POTS	POTS	POTS	POTS	POTS	POTS	**Average**	**SD**
Isoproterenol 50uM	0.156749463	0.454898057	0.547203866	0.535473182	0.721859505	0.654470213	0.664956354	0.706083948	0.424068884	0.111131615	0.366050298	0.557590795	0.610792998	0.431930458	0.226398633	0.415474108	0.373396785	0.371062565	0.625640919	0.362390636	0.442215998	**0.464754252**	**0.171447732**
Isoproterenol 5uM	0.090297889	0.414112427	0.47660783	0.462834863	0.671516265	0.562125476	0.637421806	0.633804434	0.288721715	0.087298201	0.27307881	0.423743143	0.502804132	0.384239953	0.143399631	0.305820998	0.306531483	0.240766823	0.500909953	0.212729931	0.393957502	**0.381558251**	**0.173432744**
Isoproterenol 500nM	0.021445931	0.361792246	0.137372495	0.176554384	0.473798359	0.44284943	0.49700068	0.484087662	0.147670976	0.096423315	0.231917784	0.053521969	0.128972505	0.207129942	0.118730803	0.277501163	0.218389871	0.174175227	0.29728075	0.177380132	0.241934289	**0.236472853**	**0.142098169**
Adrenaline 100uM	0.316382614	0.509721541	0.71444799	0.707350567	0.818291625	0.754337883	0.761057942	0.869731899	0.75135808	0.368299043	0.493303559	0.755200833	0.805965643	0.622678844	0.66937647	0.774445363	0.522685804	0.681461538	0.816032472	0.595512605	0.703395617	**0.667192282**	**0.149801168**
Adrenaline 10uM	0.012056469	0.346674228	0.702239757	0.445044964	0.668501249	0.53979561	0.553056092	0.70183702	0.380203719	0.048418757	0.001329274	0.381424631	0.51501055	0.396829553	0.150703741	0.29621646	0.278602623	0.177285115	0.425578171	0.159515376	0.392972665	**0.360633144**	**0.213619053**
Adrenaline 1uM	-0.015448047	0.289156028	0.235449575	0.195481761	0.430503844	0.348350595	0.383895481	0.496693975	0.146254106	-0.00631175	0.14130248	0.138728228	0.232666467	0.157691415	0.114046508	0.179366563	0.30316697	0.045122596	0.208491277	0.070120018	0.21725061	**0.205332319**	**0.135503505**

Healthy n=20	1	2	3	4	5	6	7	8	9	10	11	12	13	14	15	16	17	18	19	20		
	HAC	HAC	HAC	HAC	HAC	HAC	HAC	HAC	HAC	HAC	HAC	HAC	HAC	HAC	HAC	HAC	HAC	HAC	HAC	HAC	**Average**	**SD**
Isoproterenol 50uM	0.765834339	0.441878525	0.351527955	0.339658212	0.216537372	0.378901414	0.363962158	0.275046351	0.444080513	0.232386317	0.457090131	0.187953801	0.590448739	0.386632795	0.329940577	0.378648098	0.174056289	0.19026372	0.436990274	0.436711032	**0.36892743**	**0.143474694**
Isoproterenol 5uM	0.640778859	0.356776125	0.255737072	0.268169694	0.11356683	0.353158656	0.274707561	0.201570322	0.409015964	0.125952484	0.436872767	0.137086803	0.527037064	0.24840413	0.281509997	0.273267489	0.12365501	0.093769821	0.297223086	0.274334272	**0.2846297**	**0.141890196**
Isoproterenol 500nM	0.358900581	0.149965454	0.193664155	0.172408354	0.102309993	0.26185227	0.19620058	0.160735746	0.247487764	0.14086587	0.295521165	0.096240573	0.395715156	0.093894073	-0.112183083	0.084401075	0.083156926	-0.035875954	0.064393681	0.180115136	**0.156488476**	**0.121333635**
Adrenaline 100uM	0.761973935	0.473304779	0.50667741	0.50434465	0.458885064	0.484882429	0.585581744	0.488451652	0.470625377	0.464659037	0.618919672	0.429938921	0.675833602	0.610623129	0.651511527	0.572962475	0.309671566	0.53604966	0.632960373	0.906953043	**0.557240502**	**0.131121427**
Adrenaline 10uM	0.648400982	0.318275892	0.261932228	0.259272939	0.11102539	0.379445934	0.283346738	0.204511969	0.371972349	0.163163032	0.416766654	0.201801123	0.504962302	0.328588412	0.327484509	0.31235293	0.155093997	0.095854792	0.259758488	0.323817112	**0.296391389**	**0.132140146**
Adrenaline 1uM	0.276602468	0.127793021	0.113756327	0.127034904	0.073539919	0.359126635	0.139345302	0.097656728	0.174705576	0.094500054	0.209907722	0.041367695	0.23338231	0.170643937	0.213451736	0.07074943	0.061448326	-0.046190546	0.059337488	0.187050341	**0.139260469**	**0.092103959**

**t-test**	**DOH vs. HAC**	**POTS vs. HAC**	**OD vs. HAC**
Isoproterenol 50uM	0.324455214	0.029604247	0.07497307
Isoproterenol 5uM	0.126095898	0.028502981	0.119221515
Isoproterenol 500nM	0.279668848	0.029780066	0.099651225
Adrenaline 100uM	0.44873992	0.00830297	0.028762857
Adrenaline 10uM	0.04615918	0.126314954	0.284381618
Adrenaline 1uM	0.229271112	0.037574622	0.11175099
