# Supplementary material for: Analysis of adenylate cyclase activity in Japanese children with orthostatic dysregulation
Source: PLoS One. 2026 Apr 30;21(4):e0347431. doi: 10.1371/journal.pone.0347431 (PMC13132173; doi:10.1371/journal.pone.0347431)
Supplement: S3 Table — (PDF) [file pone.0347431.s003.pdf]

S3 Table. Raw data of of adrenaline, noradrenaline, and dopamine levels in the blood.

	OH	OH	OH	OH	POTS	POTS	OH	OH	OH	POTS	OH	POTS	POTS	POTS	POTS	POTS	POTS	POTS	INOH	POTS	POTS	POTS	POTS	POTS	POTS	POTS	POTS	POTS	POTS	POTS		
	1	2	3	4	5	6	7	8	9	10	11	12	13	14	15	16	17	18	19	20	21	22	23	24	25	26	27	28	29	30	**Average**	**Standard Deviation**
Adrenaline	19	50	18	29	35	49	36	17	28	44	36	25	35	25	25	137	127	33	15	22	14	16	15	16	13	21	37	12	20	39	**33.6**	**28.86186**
Noradrenaline	118	287	315	238	145	213	200	240	325	447	186	208	328	241	196	275	327	155	257	341	207	271	58	155	206	269	201	130	152	259	**231.6667**	**80.58379**
Dopamine	5	7	8	11	5	7	8	7	7	11	12	5	5	5	5	9	5	22	8	5	5	7	5	5	16	11	12	13	5	5	**8.033333**	**4.012767**

	**1**	**2**	**3**	**4**	**5**	**6**	**7**	**8**		
	OH	OH	OH	OH	OH	OH	OH	OH		
	1	2	3	4	7	8	9	11	**Average**	**Standard Deviation**
Adrenaline	19	50	18	29	36	17	28	36	**29.125**	**11.3696**
Noradrenaline	118	287	315	238	200	240	325	186	**238.625**	**70.15073**
Dopamine	5	7	8	11	8	7	7	12	**8.125**	**2.295181**
	1	2	3	4	5	6	7	8		

	**1**	**2**	**3**	**4**	**5**	**6**	**7**	**8**	**9**	**10**	**11**	**12**	**13**	**14**	**15**	**16**	**17**	**18**	**19**	**20**	**21**			
	POTS	POTS	POTS	POTS	POTS	POTS	POTS	POTS	POTS	POTS	POTS	POTS	POTS	POTS	POTS	POTS	POTS	POTS	POTS	POTS	POTS			
	5	6	10	12	13	14	15	16	17	18	20	21	22	23	24	25	26	27	28	29	30	**Average**	**Standard Deviation**	***t*- test**
Adrenaline	35	49	44	25	35	25	25	137	127	33	22	14	16	15	16	13	21	37	12	20	39	**36.19048**	**33.6194**	**0.202892**
Noradrenaline	145	213	447	208	328	241	196	275	327	155	341	207	271	58	155	206	269	201	130	152	259	**227.8095**	**87.3296**	**0.367035**
Dopamine	5	7	11	5	5	5	5	9	5	22	5	5	7	5	5	16	11	12	13	5	5	**8**	**4.63681**	**0.462**
